# Supplementary material for: Selective lifelong suppression of an odor processing channel in response to critical period experience
Source: eLife. 2026 Sep 28;14:RP108236. doi: 10.7554/eLife.108236 (PMC13619131; doi:10.7554/eLife.108236)
Supplement: Peer review history

# Editor report: eLife Assessment

**DOI:** 10.7554/eLife.108236.3.sa0

**Authors:** Mani Ramaswami

**Affiliations:** Trinity College Dublin Ireland

**Keywords:** Convincing, Valuable

This study in the *Drosophila* antennal lobe, which contains multiple non-equivalent sensory channels, provides **valuable** new insight into how early-life sensory experience can produce lasting, cell-type-specific changes in neural circuit function. The work demonstrates that glial-mediated pruning during a defined developmental window leads to persistent suppression of odor responses in one olfactory neuron type, while sparing another. The evidence is **convincing** and supported by multiple complementary approaches, although some mechanistic interpretations remain speculative and would benefit from additional functional testing.

# Referee report: Reviewer #1 (Public review)

**DOI:** 10.7554/eLife.108236.3.sa1

Summary:

This study builds on earlier work showing that early-life odor exposure can trigger glial-mediated pruning of specific olfactory neuron terminals in Drosophila. Moving from indirect to direct functional imaging, the authors show that pruning during a narrow developmental window leads to long-lasting suppression of odor responses in one neuron type (Or42a) but not another (Or43b). The combination of calcium and voltage imaging with connectomic analysis is a strength, though the voltage imaging results are less straightforward to interpret and may not reflect synaptic output changes alone.

Strengths:

Biologically, one of the main strengths of this work is the direct comparison between two odor-responsive OSN types that differ in their long-term adaptation to early-life odor exposure. While Or42a OSNs undergo pruning and remain persistently suppressed into late adulthood, Or43b OSNs, which also respond to the same odor, show little lasting change. This contrast not only underscores the cell-type specificity of critical-period plasticity but also points to a potential role of inhibitory network architecture in determining susceptibility. The persistence of the Or42a suppression well beyond the developmental window provides compelling evidence that early glia-mediated pruning can imprint a stable, life-long functional state on selected sensory channels. By situating these functional outcomes within the context of detailed connectomic data, the study offers a framework for linking structural connectivity to long-term sensory coding stability or vulnerability.

Comments on revised version:

I thank the authors for their careful revision and thoughtful responses to the reviewers' comments. The revised manuscript addresses my previous concerns in a satisfactory manner, and the interpretation of the findings has been appropriately clarified and balanced. I have no further major comments.

# Referee report: Reviewer #2 (Public review)

**DOI:** 10.7554/eLife.108236.3.sa2

Recent work from the authors identified the synaptic changes and glial reaction that occurs during exposure of a Drosophila odorant receptor neuron population to continued exposure of a stimulating odorant. This work markedly advanced our understanding of cellular response to critical periods. This current Advance manuscript carries that work forward and examines the non-autonomous responses to constant odorant exposure. The authors discover that the changes to ORN populations are not accompanied by changes to either PN dendrite or PN axon volume, nor are they concurrent with changes in postsynaptic PN structures. These changes are, however, notable accompanied by changes in Ca2+ and voltage responses in ORNs. Importantly, this set of responses is specific for the Or42a ORNs (that are highly sensitive to the odorant in question, ethyl butyrate) and not the Or43b ORNs (which respond to ethyl butyrate, but not as drastically). Finally, the authors include connectomics analyses showing that Or43b and Or42a ORNs differ in their synaptic input/output relationships.

This is an excellent use of the Advance mechanism for the journal as these are important follow-up findings for the parent story. The non-autonomous effects (or lack thereof) on PNs is an important part of the story as is the functional response of Or42a ORNs and the differing response of similarly (but not identically) sensitive Or43b ORNs. The experiments are well conceived, controlled, and conducted. Where the story falters a bit, though, is with the connectomics analysis. The authors show distinct differences between Or43b and Or42b ORN input output relationships and suggest that those differences may underlie the differences observed in their response to ethyl butyrate exposure during the critical period. This is certainly a possibility, but as it stands now, it is too disconnected to offer significant proof. There would have to be additional experiments to address this. Right now, the inclusion of the connectomics work feels like a distraction at best, and a complete non sequitur at worst. To be clear, the connectomics work is well done and I have no issues with its validity, but is not helpful to the central thesis of the work. I would suggest the authors either remove it entirely or strongly rethink how it fits into the paper.

Comments on revised version:

I appreciate the consideration of my comments and the authors' responses. The additional data on PN synapse number is intriguing (and welcome) as is the text discussing potential postsynaptic compensatory mechanisms. I respect the authors' decision in retaining the connectivity analysis, but despite the textual changes, I still feel that it is peripherally related to the main thesis of the work and would best be omitted from the paper and included in a separate, more relevant study. Ultimately, though, that is their choice.

# Author comment: Author response

**DOI:** 10.7554/eLife.108236.3.sa3

**Authors:** Hans C Leier, Julius Jonaitis, Alexander J Foden, Abigail J Wilkov, Paola Van der Linden Costello, Heather Broihier, Andrew M Dacks

**Affiliations:** Case Western Reserve University School of Medicine Cleveland United States; Case Western Reserve University Cleveland United States; Case Western Reserve Unversity School of Medicine Cleveland United States; Case Western Reserve University School of Medicine Cleveland United States; Case Western Reserve University School of Medicine Cleveland United States; Case Western Reserve University Cleveland United States; Case Western Reserve University Cleveland United States

The following is the authors’ response to the original reviews.

**Public Reviews:**

**Reviewer #1 (Public review):**

Summary:

This study builds on earlier work showing that early-life odor exposure can trigger glial-mediated pruning of specific olfactory neuron terminals in Drosophila. Moving from indirect to direct functional imaging, the authors show that pruning during a narrow developmental window leads to long-lasting suppression of odor responses in one neuron type (Or42a) but not another (Or43b). The combination of calcium and voltage imaging with connectomic analysis is a strength, though the voltage imaging results are less straightforward to interpret and may not reflect synaptic output changes alone.

Strengths:

Biologically, one of the main strengths of this work is the direct comparison between two odor-responsive OSN types that differ in their long-term adaptation to early-life odor exposure. While Or42a OSNs undergo pruning and remain persistently suppressed into late adulthood, Or43b OSNs, which also respond to the same odor, show little lasting change. This contrast not only underscores the cell-type specificity of critical-period plasticity but also points to a potential role of inhibitory network architecture in determining susceptibility. The persistence of the Or42a suppression well beyond the developmental window provides compelling evidence that early glia-mediated pruning can imprint a stable, life-long functional state on selected sensory channels. By situating these functional outcomes within the context of detailed connectomic data, the study offers a framework for linking structural connectivity to long-term sensory coding stability or vulnerability.

Weaknesses:

The narrative begins with the absence of changes in PN dendrites and axons. While this establishes specificity, it is a relatively weak starting point compared to the novel OSN functional results.

We agree that switching the order of Figures 1 and 2 recontextualizes the negative PN morphology findings to make their significance more clear, especially with the addition of PN odour-evoked activity data (see Figures 2A, B of the revised manuscript).

Calcium imaging with GCaMP, though widely used, is an indirect measure of synaptic function, and reduced signals could reflect changes in non-synaptic calcium influx as well as release probability. The interpretation of the voltage imaging results is also unclear: if suppression were solely due to impaired synaptic release, one might expect action potential-evoked voltage signals to remain unchanged. The reported changes raise the possibility of deficits in action potential initiation or propagation, which would shift the mechanistic explanation.

Although it is true that non-synaptic Ca^2+^ influx could contribute to odour-evoked signals in OSN axon terminals, it seems likely to be a relatively small contribution when compared to Ca^2+^ influx via voltage-gated Ca^2+^ channels at the active zone. Given the observation that synaptic markers are eliminated during this form of critical period plasticity and remain decreased even after OSNs regrow their terminals days later (consistent with our observed continued decrease in odour-evoked responses), the most parsimonious explanation is that we are seeing a reduction in synaptic Ca^2+^ influx. We cannot dismiss the possibility that there is a decreased voltage signal arising from fewer action potentials being elicited by the odour stimulation. However, the reduction in voltage signal must arise at least in part from the observed reduction in Ca^2+^ influx. We have therefore provided additional text to this effect in the results section.

The difference between Or42a and Or43b OSNs is attributed to varying inhibitory input densities from connectome data, but this remains speculative without functional tests such as manipulating GABA receptor expression in OSNs. In Or43b, there is essentially no strong phenotype, making it premature to ascribe the absence of suppression solely to inhibitory connectivity.

We have tempered our conclusions to posit additional mechanisms that could explain the more mild pruning that occurs for Or43b OSNs. While the pruning phenotype for Or43b OSNs is not as strong as Or42a, it is not absent. To further explore the contribution of inhibition as a candidate mechanism underlying differences in susceptibility of Or42a and Or43b to this form of critical period plasticity we compared the relative impact of knocking down expression of GABA-A receptor (called “*rdl*”) in Or42a and Or43b OSNs. Consistent with the degree of pruning being regulated inhibition, knocking down expression of *rdl* enhanced pruning for both Or42a and Or43b OSNs. However, because the magnitude of the enhancement was similar between both OSN types, we agree with the reviewer that inhibitory connectivity cannot be the sole mechanism that explains the difference and have therefore tempered our language appropriately.

Finally, the study does not connect circuit-level changes to behavioral outcomes; assays of odor-guided attraction or discrimination could place the findings in an organismal context.

We agree that behavioral assays will be a critical component for understanding the functional consequences of this form of critical period plasticity. However, the goal of this study was to extend our prior work to determine the longevity and selectivity of the critical period pruning. Behavioral assays testing the consequences of this form of early life plasticity will be a component of future studies.

Some introduction material overlaps with the authors' 2024 paper, and the novelty of the present study could be signposted more clearly.

We have included text to highlight the novelty of the present study.

**Reviewer #2 (Public review):**

Recent work from the authors identified the synaptic changes and glial reaction that occur during exposure of a Drosophila odorant receptor neuron population to continued exposure of a stimulating odorant. This work markedly advanced our understanding of cellular response to critical periods. This current Advance manuscript carries that work forward and examines the non-autonomous responses to constant odorant exposure. The authors discover that the changes to ORN populations are not accompanied by changes to either PN dendrite or PN axon volume, nor are they concurrent with changes in postsynaptic PN structures. These changes are, however, notable, accompanied by changes in Ca2+ and voltage responses in ORNs. Importantly, this set of responses is specific to the Or42a ORNs (that are highly sensitive to the odorant in question, ethyl butyrate) and not the Or43b ORNs (which respond to ethyl butyrate, but not as drastically). Finally, the authors include connectomics analyses showing that Or43b and Or42a ORNs differ in their synaptic input/output relationships.

This is an excellent use of the Advance mechanism for the journal, as these are important follow-up findings for the parent story. The non-autonomous effects (or lack thereof) on PNs is an important part of the story, as is the functional response of Or42a ORNs and the differing response of similarly (but not identically) sensitive Or43b ORNs. The experiments are well-conceived, controlled, and conducted. Where the story falters a bit, though, is with the connectomics analysis. The authors show distinct differences between Or43b and Or42b ORN input-output relationships, and suggest that those differences may underlie the differences observed in their response to ethyl butyrate exposure during the critical period. This is certainly a possibility, but as it stands now, it is too disconnected to offer significant proof. There would have to be additional experiments to address this. Right now, the inclusion of the connectomics work feels like a distraction at best, and a complete non sequitur at worst. To be clear, the connectomics work is well done and I have no issues with its validity, but it is not helpful to the central thesis of the work. I would suggest the authors either remove it entirely or strongly rethink how it fits into the paper.

We have tempered our stated interpretations of the connectivity analysis and include new experiments examining the impact of GABA signaling on pruning. We have therefore opted to retain the connectivity analysis as we feel that it has been better integrated into the overall narrative of the paper.

Major Concerns:

(1) The examination of PN axon terminals in the MB and LH is interesting, but it is only one possibility. Oftentimes, the volume of neurons remains constant with perturbation, while the synapse number is affected. Figure 1C and E would be greatly helped by examining synapse number (via Brp or Brp-Short) in the PN axons.

We agree that the counting synapse number would provide greater resolution information about synapse function relative to axon volume and have added this analysis to what is now Figure 2.

(2) The use of dlg1[4K] is a strong use of a new tool, but the result is surprising. The presynaptic ORN synapse number onto the PNs is notably changed, but that is not reflected in a postsynaptic PSD-95 change. That suggests a compensatory mechanism that the authors might explore. A good proportion of PN puncta should be postsynaptic to those ORNs, so why aren't they adjusted?

We agree that this result suggests that a compensatory mechanism may be present. We have therefore added new text to point out this observation and potential explanation.

**Recommendations for the authors:**

**Reviewer #1 (Recommendations for the authors):**

The interpretation of the voltage imaging results would benefit from clarification. If these signals are reduced because of upstream action potential changes rather than synaptic release, this should be explicitly discussed and illustrated with representative raw traces for both OSN types. The proposed link between inhibitory connectivity and selective vulnerability could be tested more directly, for example, by manipulating GABA receptor function in OSNs.

We have now tested the link between inhibitory connectivity and susceptibility to glial pruning by testing the effects of GABA receptor knockdown in either Or42a or Or43b OSNs (fully described above).

Adding an intermediate post-exposure time point for Or42a responses could help resolve whether suppression is immediate or develops over time.

The suppression of Or42a odour-evoked responses is present immediately after the 2 day exposure period and responses remain suppressed until 25 days post-eclosion, indicating that the suppression is immediate and sustained. We therefore respectfully disagree that another physiological time point will help resolve whether the suppression is immediate or develops over time.

In terms of presentation, the introduction could be tightened to reduce overlap with the 2024 paper, figures should have clear axis labels and consistent terminology for neuron types and glomeruli, and a schematic summarising key inhibitory connections for Or42a vs. Or43b would aid clarity

We have now streamlined the introduction, improved clarity on axis labels and checked for consistency of terminology.

Minor Concerns:

(1) The dlg1[4K] is made with a V5 epitope but the authors have it labeled mCD8::GFP in Figure 1F. This is likely a typo and should be corrected.

This typo has now been corrected.

(2) Can the responses be separated in Figures 2A, C, and E? It is difficult to see the differences in oil and EB exposure. This would make it much more straightforward to tell the difference if both traces were clearly visible.

Overlaying the averaged response traces for in Figure 2C, E and G (now Figures 1C, E and G) enables the reader to make direct visual comparisons between the responses of OSNs from flies in each condition to both mineral oil and ethylbutyrate. Separating the individual traces would make it much more difficult to make these comparisons.
